# Reduced myotube diameter induced by combined inhibition of transforming growth factor‐β type I receptors *Acvr1b* and *Tgfbr1* is associated with enhanced β1‐syntrophin expression

**DOI:** 10.1002/jcp.31418

**Published:** 2024-08-20

**Authors:** Andi Shi, Chuqi He, Kirsten Otten, Gang Wu, Tymour Forouzanfar, Rob C. I. Wüst, Richard T. Jaspers

**Affiliations:** ^1^ Laboratory for Myology, Department of Human Movement Sciences Faculty of Behavioural and Movement Sciences, Vrije Universiteit Amsterdam, Amsterdam Movement Sciences Amsterdam The Netherlands; ^2^ Guangzhou Key Laboratory of Basic and Applied Research of Oral Regenerative Medicine, Department of Prosthodontics Affiliated Stomatology Hospital of Guangzhou Medical University, Guangdong Engineering Research Center of Oral Restoration and Reconstruction Guangzhou China; ^3^ Department of Oral Cell Biology Academic Centre for Dentistry Amsterdam (ACTA), University of Amsterdam (UvA) and Vrije Universiteit Amsterdam (VU) Amsterdam The Netherlands; ^4^ Department of Oral and Maxillofacial Surgery Leiden University Medical Center Leiden The Netherlands

**Keywords:** Acvr1b, myotubes, Tgfbr1, TGF‐β type I receptor, β1‐syntrophin

## Abstract

Simultaneous inhibition of transforming growth factor‐β (TGF‐β) type I receptors *Acvr1b* and *Tgfbr1* signalling has been associated with excessive skeletal muscle hypertrophy in vivo. However, it remains unclear whether the increased muscle mass in vivo is a direct result of inhibition of intracellular TGF‐β signalling or whether this is an indirect effect of an altered extracellular anabolic environment. Here, we tested whether individual or simultaneous knockdown of TGF‐β type I receptors in C2C12 myotubes was sufficient to induce muscle hypertrophy. The expression levels of TGF‐β type I receptors *Acvr1b* and *Tgfbr1* in myotubes were knocked down individually or in combination in the absence or presence of TGF‐β1 and myostatin. Knocking down either *Acvr1b* or *Tgfbr1* did not significantly change cell phenotype. Unexpectedly, simultaneous knockdown of both receptors reduced C2C12 myotube diameter, mRNA expression levels of *Hgf*, *Ccn2* and *Mymx* with or without TGF‐β1 and myostatin administration. In spite of decreased phosphorylation of Smad2/3, phosphorylation of P70S6K was reduced. In addition, the gene expression level of β1‐syntrophin (*Sntb1*), which encodes a protein associated with the dystrophin−glycoprotein complex, was increased. Parallel experiments where *Sntb1* gene expression was reduced showed an increase in myotube diameter and fusion of C2C12 myoblasts. Together, these results indicate that the knockdown of both TGF‐β type I receptors reduced myotube diameter. This atrophic effect was attributed to reduced protein synthesis signalling and an increased expression of β1‐syntrophin. These results have implications for our fundamental understanding of how TGF‐β signalling regulates skeletal muscle size.

## 1 INTRODUCTION

Skeletal muscle wasting diseases cause progressive loss in muscle mass and strength, eventually resulting in muscle atrophy. Muscle atrophy is associated with ageing, physical inactivity and pathophysiological conditions, such as Duchenne's muscular dystrophy (DMD) and cancer cachexia. Muscle atrophy worsens quality of life and increases mortality (Aquila et al., 2020; Vinciguerra et al., 2010). In addition to muscle atrophy, muscle fibrosis and degeneration are hallmarks of ageing and DMD (Kharraz et al., 2014; Tidball et al., 2021). Muscle‐wasting diseases have been shown to be associated with excessive expression of transforming growth factor‐β (TGF‐β) superfamily members, including TGF‐β isoforms, myostatin and activin A (Klein, 2022; Rodriguez et al., 2014; Thissen & Loumaye, 2013). Myostatin is a critical negative regulator for muscle mass (Bataille et al., 2021) and excessive expression of TGF‐β1 leads to muscle fibrosis and degeneration (Mazala et al., 2020). Considerable efforts have been made to target TGF‐β signalling as a potential treatment for muscle wasting disorders (Leung & Wagner, 2013; Ohsawa et al., 2012).

TGF‐β signalling is initiated by binding of ligands to the activin receptor type II or the TGF‐β receptor type II (TβRII). Upon binding to ligands, type II receptors form heterotetramers with TGF‐β and activin type I receptors to phosphorylate transcriptional factors Smad2/3 and block protein kinase B (AKT)/mammalian target of rapamycin (mTOR) pathway, resulting in reduced protein synthesis and consequently leading to muscle atrophy (Tzavlaki & Moustakas, 2020). Although TGF‐β and myostatin are structurally similar, they bind to different type I receptors. In skeletal muscle, TGF‐β isoforms bind to TGF‐β receptor type I (TGFBR1, ALK5) (Heldin & Moustakas, 2016), while myostatin binds to both TGFBR1 and activin receptor type IB (ACVR1B, ALK4) (Kemaladewi et al., 2012; Rebbapragada et al., 2003). Targeting TGF‐β ligands by administration of myostatin and activin antagonists induced fast‐type muscle hypertrophy by 150% within 8 weeks (Chen et al., 2017). An even more promising approach to induce muscular hypertrophy and potentially increase muscle strength seems to be the interference with type I receptors of the TGF‐β superfamily rather than targeting the ligands (Lee et al., 2020). Simultaneous myofibre‐specific knockout of type I receptors of the TGF‐β family after 10 weeks increased muscle mass in fast‐type mice gastrocnemius medialis muscle by ~200%, while knockout of type II receptors only increased muscle mass by ~63% (Lee et al., 2020). In addition to hypertrophy, fast‐type muscles also show regions of regenerating myofibres with concomitant infiltration of macrophages in fast‐type myofibre‐dominated regions (Hillege et al., 2022). These changes are accompanied by increased expression levels of hepatocyte growth factor (*Hgf*), transforming growth factor beta (*Tgfb1*), myostatin (*Mstn*) and decreased expression levels of mechano growth factor (*Mgf*) and vascular endothelial growth factor (*Vegfa*). Since HGF stimulates the AKT/mTOR signalling pathway (Perdomo et al., 2008), enhanced expression of HGF likely contributed to the hypertrophic response in fast‐type myofibres. However, it is unclear whether the hypertrophy of fast‐type muscle is a direct effect of knocking out the TGF‐β type I receptors or depends on the biochemical composition in the interstitial space. We hypothesized that non‐muscle cells or growth factors, such as HGF, are indispensable for TGF‐β type I receptor knockout‐induced muscle hypertrophy. To investigate the direct effect of signalling via one or both of the TGF‐β type I receptors and avoid interaction effects from non‐muscle cells, cultured myotubes were treated with siRNA targeting *Acvr1b* and *Tgfbr1* to inhibit intracellular TGF‐β/myostatin signalling. Moreover, muscle cells were treated with TGF‐β1 and myostatin to mimic the in vivo conditions of atrophic muscle.

The increases in muscle mass and maximal force have been shown to be greater in fast‐type muscle than in slow‐type muscle of myostatin‐null mice (Mendias et al., 2006). While myofibre‐specific knockout of both TGF‐β type I receptors in fast‐type gastrocnemius medialis muscle reduced specific muscle force by 22% compared to control muscle 3 months after gene knockout, indicating that the increase in strength was not proportional with the increase in muscle mass (Shi et al., 2023). In contrast, the slow‐type soleus muscles showed a smaller amount of increase in muscle mass and a proportional increase in strength (Mendias et al., 2006). These results raise the question, what are the underlying mechanisms of the differential specific force between fast‐and slow‐type muscles with myofibre‐specific TGF‐β type I receptors knockout. A possible mechanism could be that the stronger anabolic effect and concomitant hypertrophy precede the energy demand to construct the T‐tubular system and the excitation‐contraction system. However, a delay does not seem to be likely as 3 months after the start of knockout, body mass has reached a plateau (Shi et al., 2023). β1‐syntrophin is a motif of the dystrophin−glycoprotein complex (DGC), which is located in the sarcolemma. It connects cytoskeleton to extracellular matrix and is involved in myofascial force transmission (Gumerson & Michele, 2011; Ramaswamy et al., 2011). Muscle Transcriptome profiling has shown that knockout causes a reduction in the expression of β1‐syntrophin (*Sntb1*) solely in fast‐type muscle (Shi et al., 2023). Reduced *Sntb1* level may explain the lower specific force of the fast‐type muscle. However, whether the TGF‐β signalling blockade‐induced hypertrophy of fast‐type muscle is associated with decreased expression of *Sntb1* is unknown.

The aim of this study was to test the direct and acute effects of downregulation of TGF‐β type I receptors on myotube size, cytokines expression and the impact of β1‐syntrophin on myotube size in vitro. To this end, *Acvr1b* and *Tgfbr1* were knocked down individually or simultaneously in myotubes in the presence or absence of TGF‐β1 or myostatin in well‐defined conditions.

## 2 MATERIALS AND METHODS

### 2.1 C2C12 cell culture

**Figure 1:**
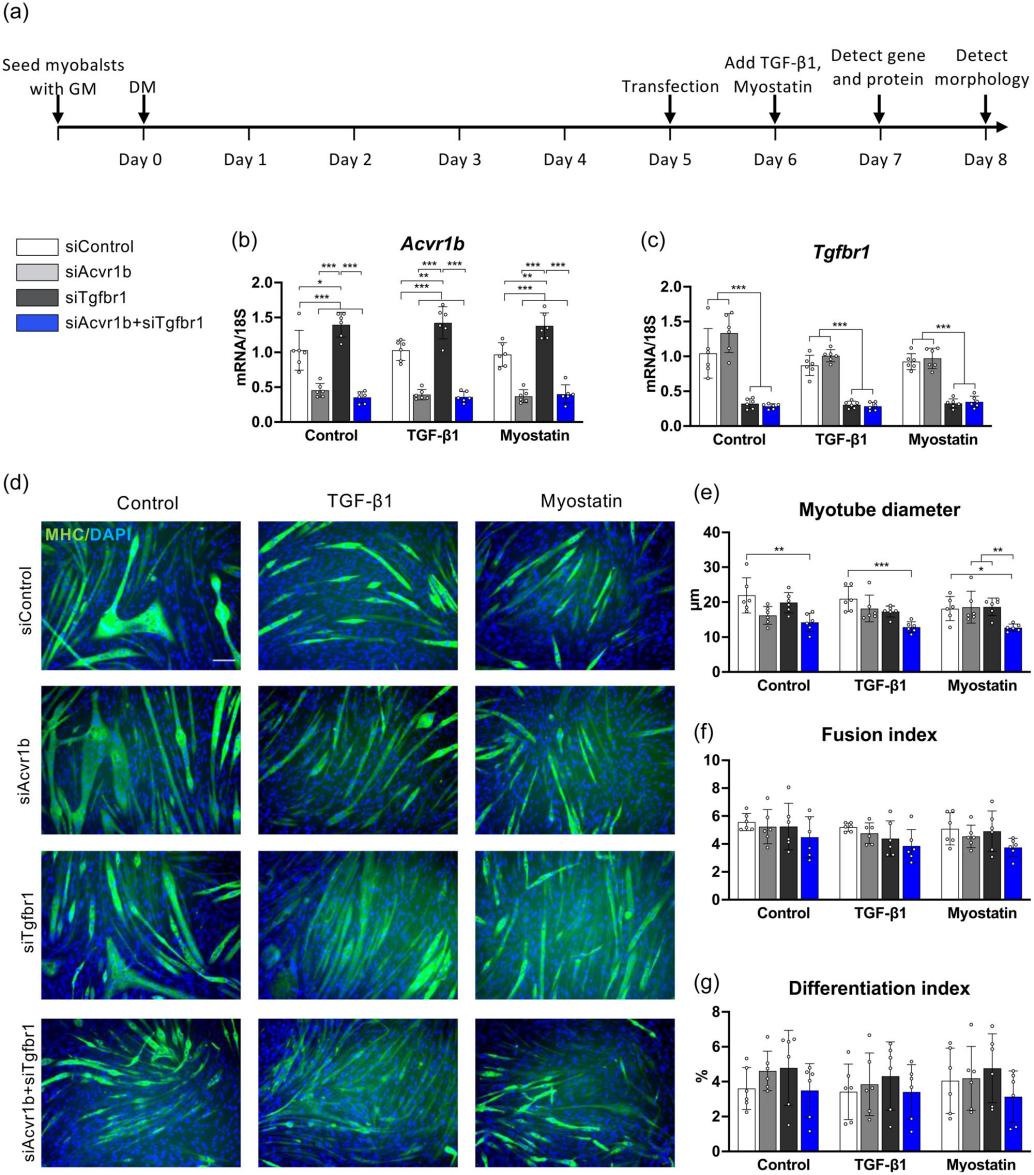
Knockdown of both TGF‐β type I receptors impairs myotube growth. (a) Scheme of siRNA assay. Myotubes were cultured in differentiation medium for 5 days and transfected by siAcvr1b and/or siTgfbr1 and a control siRNA (siControl) for 3 days. Expression levels of (b) *Acvr1b* and (c) *Tgfbr1* in myotubes transfected by siRNA for 48 h. (d) Immunofluorescent staining of myosin heavy chain (MHC) (green) reveals reduced myotube size in siAcvr1b + siTgfbr1 groups. Nuclei were stained by DAPI (blue). Scale bar = 100 µm. (e) Myotube growth was impaired in siAcvr1b + Tgfbr1 groups compared with siControl in the presence or absence of TGF‐β1 or myostatin. Both the (f) fusion index and (g) differentiation index were not different between groups in myotubes treated upon transfection. Data are presented as mean ± SD. *p < 0.05, **p < 0.01, ***p < 0.001. *N* = 6. Data were analysed by three‐way ANOVA (individual factors: siAcvr1b, siTgfbr1 and cytokines). Post hoc analysis was performed by Bonferroni corrections. TGF‐β, transforming growth factor‐β.

C2C12 mouse myoblast cell line (ATCC, CRL‐1772) was obtained from ATCC and cells were grown to confluency in growth medium consisting of Dulbecco's Modified Eagle's Medium (11995065; Gibco), 10% foetal bovine serum (Biowest; S181B), 1% penicillin/streptomycin (Gibco; 15140‐122) and 0.5% amphotericin B (Gibco; 15290‐026) and were incubated at 37°C in humidified air with 5% CO_2_. Plates were coated with collagen (collagen I rat protein, tail (Gibco; A10483‐01) diluted in 0.02 N acetic acid). Once cells met 90% confluence, the growth medium was changed into differentiation medium for 4 growing days containing DMEM supplemented with 2% horse serum (HyClone; 10407223) and 1% penicillin/streptomycin, 0.5% amphotericin B. Medium was refreshed every other day. After 4 days of differentiation, the medium was changed into an antibiotic‐free differentiation medium for 24 h before transfection. Twenty‐four hours after transfection with siAcvr1b, siTgfbr1, siSntb1 and negative siRNA (Table 1), cells were supplemented with 10 ng/mL TGF‐β1 (Peprotech; 100‐21C) or 300 ng/mL myostatin (Peprotech; 120‐00) as experimental groups or with vehicle as control, based on previous experiments (Hillege et al., 2020). For gene and protein expression analyses, cells were lysed 48 h after transfection. For immunofluorescence staining, cells were fixed 72 h after transfection (Figure 1a). After siSntb1 transfection, C2C12 myotubes were lysed or fixed 24, 48 and 72 h later to assess gene and protein expression levels and myotube diameter (Figure 3a).

**Table 1:** Sequence of siRNA.

Silenced gene	Forward	Reverse
*Acvr1b*	GACUUGAAGUCAAAGAACATT	UGUUCUUUGACUUCAAGUCTC
*Tgfbr1*	GACUAUCAGUUGCUUAUUtt	AAUAAGGCAACUGAUAGUCTT
*Sntb1*	AGCCGAUCGUUUUCAUCAUTT	AUGAUGAAAACGAUCGGCUTG
Negative	AGUACUGCUUACGAUACGGTT	CCGUAUCGUAAGCAGUACUTT

### 2.2 siRNA‐mediated knockdown of *Acvr1b*, *Tgfbr1* and *Sntb1*

To carry out an ACVR1B and TGFBR1 loss‐of‐function experiment, *Acvr1b* and *Tgfbr1* expression levels in C2C12 myotubes were knocked down using small interference RNA (siRNA). C2C12 myotubes were grown as described. After 5 days in DM, cells were transfected with siRNA targeting *Acvr1b* (Ambion; s61929) and *Tgfbr1* (Ambion; s75061) individually or simultaneously or *Sntb1* (Ambion; s202017) using the liposome‐mediated method (Lipofectamine^TM^ RNAiMAX Reagent [Invitrogen; 13778‐030]). As a negative control, a nontargeting silence RNA sequence (Invitrogen; 4390843) was used (Table 1). siRNA was diluted in Opti‐MEM medium (Opti‐MEM® I Reduced‐Serum Medium; 31985‐070) and incubated for 5 min at room temperature with Lipofectamine mixture. Then, cells were cultured in antibiotic‐free DM with 160 nM siNegative (siControl group), 80 nM siAcvr1b + 80 nM siControl (siAcvr1b group), 80 nM siTgfbr1 + 80 nM siControl (siTgfbr1 group), 80 nM siAcvr1b + 80 nM siTgfbr1 (siAcvr1b+siTgfbr1 group) or 50 nM siNegative (siControl) and 50 nM siSntb1 (siSntb1 group).

### 2.3 Immunofluorescence staining

Myotube size was assessed by immunofluorescence staining. Myotubes were fixed with 4% paraformaldehyde 72 h post‐transfection. Cells were washed 5 min with PBS and permeabilised by 0.5% Triton X‐100 in PBS for 5 min. After washing 3 × 5 min in PBS with 0.025% Tween 20 (PBS‐T), cells were blocked by 5% normal goat serum (ThermoFisher Scientific; 50062Z) for 1 h at room temperature. Subsequently, cells were incubated overnight with myosin monoclonal antibody MF‐20 (Developmental Studies Hybridoma Bank, University of Iowa; 1:50). Cells were washed 3 × 5 min and incubated for 1 h with secondary antibody Alexa Fluor 488 goat anti‐mouse conjugate (A‐21141 ThermoFisher; 1:1000). After 3 × 5 min washes in PBS‐T, mounting medium with DAPI was used to stain nuclei (VECTASHIELD, H‐1200, Vector Laboratories). Images were taken with a Zeiss fluorescence microscope and a CCD camera. Images were analysed with ImageJ (National Institutes of Health; RRID:SCR_003070) to obtain the fusion index, differentiation index and myotube diameter. Myotubes should contain at least two nuclei (Hinkle et al., 2021). The fusion index was defined as the total number of nuclei within myotubes divided by the total number of myotubes. The differentiation index was defined as the total number of myotubes divided by the total number of cells. The diameters of 20 myotubes were measured to assess the mean myotube diameter in every well.

### 2.4 RNA isolation and reverse transcription

After washing C2C12 cells with PBS, cells were lysed in a TRIzol reagent (Invitrogen; 11312940). RNA was isolated using a RiboPure kit (Thermo Fisher Scientific; AM1924) and 500 ng RNA was converted to cDNA with high‐capacity RNA to cDNA master mix (Applied Biosystems; 43889850). cDNA was 10 times diluted with RNAse‐free water for real‐time quantitative PCR for all genes, apart from cDNA for the 18S housekeeping gene that was 1000 times diluted.

### 2.5 Real‐time quantitative PCR

cDNA was analysed using real‐time quantitative PCR (Supporting Information S1: Table 1). Experiments were conducted in duplicates. Relative expression levels of the transcriptional targets were detected with fluorescent SYBR Green Master Mix (Thermo Fisher Scientific; A25742). Transcriptional expression levels of the target genes were normalized to 18S rRNA. Relative changes in gene expression were determined with the Δ*C*_t_ method, using the StepOne real‐time PCR (Applied Biosystems).

### 2.6 Western blot analysis

Cells were lysed within RIPA buffer (Sigma‐Aldrich; R0278) containing one tablet of protease inhibitor (Sigma‐Aldrich; 11836153001) and one tablet of phosStop (Sigma‐Aldrich; 04906837001) per 10 mL. The total protein concentration in the lysates was determined using a Pierce BCA Protein Assay kit (Thermo Scientific; 23225). Respective volumes of lysates were diluted in three times Laemmli SDS buffer and denatured for 5 min at 95°C, before western blot analysis. A 4%−20% Mini‐PROTEAN® TGX™ Precast Protein Gels was used and proteins were transferred onto PVDF membranes (GE Healthcare; Cat#15269894) at 80 V for 60 min. The membrane was incubated for 1 h at room temperature in 2% enhanced chemiluminiscence prime blocking agent (RPN418; GE Healthcare). Membranes were incubated overnight at 4°C in a blocking buffer with primary antibody (Supporting Information S1: Table 2). Incubation with a secondary antibody was done for 1 h at RT in a blocking buffer the other day, and detection was performed using an ECL detection kit (RPN2235; GE Healthcare). Images were taken by the ImageQuant LAS500 (GE Healthcare, Life Sciences) and the relative intensity of protein bands were quantified using ImageJ (NIH).

### 2.7 Quantification of protein synthesis rate

Myotubes were cultured according to the schematic protocol shown in Figure 4a. To quantify protein synthesis rate of myotubes, a puromycin incorporation SUnSET assay was performed (Ravi et al., 2020). 1 μM puromycin was added to differentiation medium and incubated for 30 min before cell lysis. Puromycin was quantified by western blot analysis.

### 2.8 Statistical analysis

Shapiro−Wilk tests were used to test for normal distribution. Three‐way ANOVA was used for normally distributed data for groups treated with siAcvr1b or/and siTgfbr1 in the presence or absence of TGF‐β1 or myostatin. Between groups factors were siAcvr1b, siTgfbr1 and cytokines. If an interaction or main effect was present, post hoc tests were performed with Bonferroni correction. A logarithmic transformation was performed if data was not normally distributed. Kruskal−Wallis tests were performed if data were not normally distributed after a logarithmic transformation. Differences between groups were considered significant if *p* < 0.05. All data analysis was performed using IBM SPSS Statistics of version 27.0 (IBM Corp.). Results were presented as mean ± SD with individual points.

## 3 RESULTS

### 3.1 Impaired myotube growth by simultaneous knockdown of *Acvr1b* and *Tgfbr1*

TGF‐β type I receptors *Acvr1b* and *Tgfbr1* were knocked down in C2C12 myotubes by siRNA targeting *Acvr1b* and *Tgfbr1* individually or simultaneously. Twenty‐four hours after transfection, myotubes were treated with or without TGF‐β1 or myostatin for 24 h (Figure 1a). The knockdown efficiency of *Acvr1b* and *Tgfbr1* was confirmed by qPCR (Figure 1b,c). Knockdown of *Acvr1b* did not increase *Tgfbr1* expression (*p* < 0.001), while knockdown of *Tgfbr1* significantly increased *Acvr1b* expression by 1.4‐fold in the absence (*p* = 0.013) or presence of TGF‐β1 (*p* = 0.001) or myostatin (*p* = 0.001).

Myotubes were stained by myosin heavy chain (MHC) antibody 48 h after transfection (Figure 1a,d). Myotube diameter was significantly reduced in siRNA‐mediate knockdown of *Acvr1b* and *Tgfbr1* compared to control in the absence (*p* = 0.005) or presence of TGF‐β1 (*p* < 0.001) or myostatin (*p* = 0.014) (Figure 1e). The fusion index and differentiation index were not significantly different between groups (Figure 1f,g).

### 3.2 Knockdown of *Acvr1b* and *Tgfbr1* downregulates Smad2/3 and P70S6K phosphorylation

**Figure 2:**
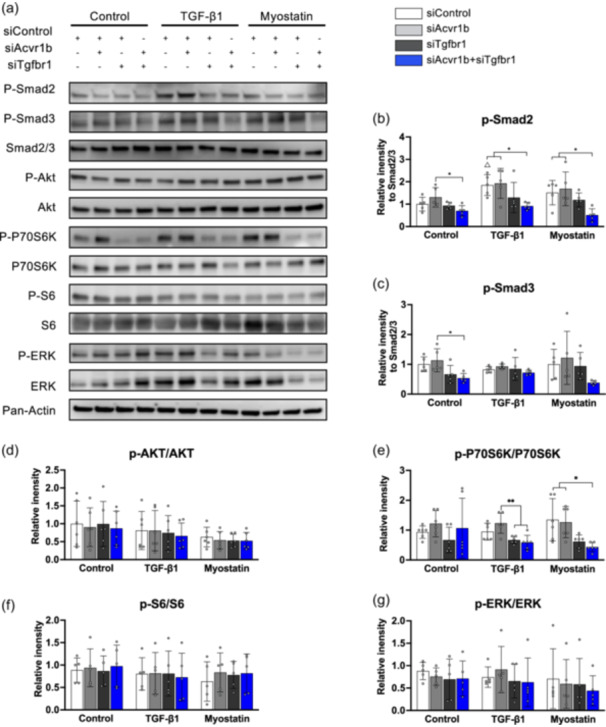
Targeting both TGF‐β type I receptors in myotubes reduces protein synthesis signalling in a Smad2/3‐independent manner. (a) Western blot analysis of proteins related to TGF‐β signalling and protein synthesis signalling pathways 48 h after transfection. (b) Relative p‐Smad2 and (c) relative p‐Smad3 were both decreased in double knockdown groups in the absence of TGF‐β1 or myostatin treatment, while only relative p‐Smad2 was decreased in double knockdown groups with either TGF‐β1 or myostatin treatment. (d) P‐AKT/AKT levels were not different between groups, while (e) those of p‐P70S6K/P70S6K after knockdown of both receptors were decreased compared to those in myotubes treated by siAcvr1b within the presence of TGF‐β1 and compared to those of the control group, as well as to those in myotubes treated by siAcvr1b in the presence of myostatin. (f) Phosphorylation of S6, a downstream kinase of the AKT/mTOR signalling pathway, was not altered by siRNA. (g) Phosphorylation of ERK1/2 in myotubes was not affected by either siAcvr1b or siTgfbr1 with or without TGF‐β1 or myostatin compared to siControl. Data are presented as mean ± SD. *p < 0.05, **p < 0.01, ***p < 0.001. *N* = 5. ^Δ^ *p* < 0.05 compared to siControl group without cytokines supplementation. Data were analysed by three‐way ANOVA (independent factors: siAcvr1b, siTgfbr1 and cytokines) with post hoc Bonferroni corrections. mTOR, mammalian target of rapamycin; TGF‐β, transforming growth factor‐β.

To investigate whether impaired myotube growth was caused by inhibition of TGF‐β signalling or ribosomal protein synthesis as well, phosphorylation levels of Smad2/3 and AKT/P70S6K/S6 signalling were analysed (Figure 2a). Relative p‐Smad2 expression levels were significantly decreased by siRNA‐mediated knockdown of both Acvr1b and Tgfbr1 compared to control in the presence of TGF‐β1 (*p* = 0.014) or myostatin (*p* = 0.041). Relative p‐Smad3 expression levels were only decreased in myotubes by knockdown of both receptors compared to myotubes exposed to siAcvr1b in the absence of TGF‐β1 or myostatin treatment (*p* = 0.027) (Figure 2b,c). Although p‐AKT/AKT ratios were not significantly different between groups by knockdown of both receptors, phosphorylation of the downstream effector P70S6K was significantly decreased in the presence of TGF‐β1 (*p* = 0.003) compared to that in myotubes treated with siAcvr1b, and compared to that in myotubes of the control group (*p* = 0.014) as well as compared to that in myotubes treated with siTgfbr1 (*p* = 0.026) in the presence of myostatin (Figure 2d,e). Nevertheless, phosphorylation of S6, the downstream target of P70S6K, was not changed by knocking down the receptors (Figure 2f). Since activation of both canonical and noncanonical TGF‐β pathway signalling is required to reduce myotube diameter (Abrigo et al., 2018), phosphorylation status of TGF‐β noncanonical pathway was assessed. Phosphorylation of ERK1/2 was not affected by knockdown of *Acvr1b* or *Tgfbr1* (Figure 2g).

### 3.3 Blocking both TGF‐β type I receptors reduces gene expression associated with muscle growth, pro‐fibrosis and fusion

**Figure 3:**
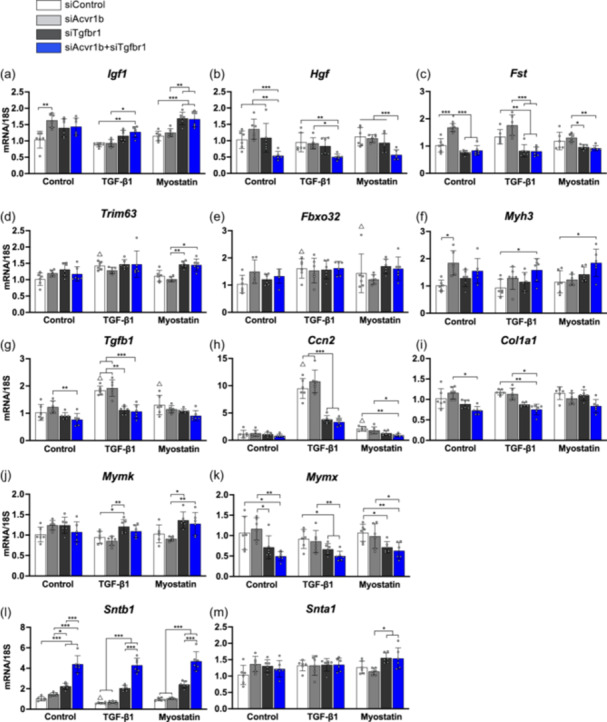
Expression levels of genes that are related to protein turnover, cell differentiation, fibrogenesis and cytoskeleton in myotubes treated with siAcvr1b or/and siTgfbr1. Gene expression levels of (a) *Igf1*, (b) *Hgf*, (c) *Fst*, (d)*Trim63*, (e) *Fbxo32*, (f) *Myh3*, (g) *Tgfb1*, (h) *Ccn2*, (i) *Col1a1*, (j) *Mymk*, (k) *Mymx*, (l) *Sntb1* and (m) *Snta1.* Data are presented as mean ± SD. *p < 0.05, **p < 0.01, ***p < 0.001. ^Δ^ *p* < 0.05 compared to siControl group without cytokines supplementation. *N* = 6. Data were analysed by three‐way ANOVA (independent factors: siAcvr1b, siTgfbr1 and cytokines) with post hoc Bonferroni corrections.

We next determined the effects of siRNA‐mediated targeting of type I receptors on gene expression levels of myotubes relative to protein turnover. Knockdown of *Acvr1b* and *Tgfbr1* caused modest increase in gene expression of insulin‐like growth factor 1 (*Igf1*) in myotubes treated with TGF‐β1 (*p* = 0.006) or myostatin (*p* < 0.000) (Figure 3a), while decreased hepatocyte growth factor (*Hgf*) expression level by about 50% in myotubes without (*p* = 0.003) or with either TGF‐β1 (*p* = 0.005) or myostatin (*p* = 0.001) (Figure 3b). Expression levels of follistatin (*Fst*), an antagonist of activin and myostatin, were decreased by transfecting myotubes with siRNA targeting *Tgfbr1* (*p* = 0.004) or *Acvr1b* and *Tgfbr1* (*p* = 0.003) with the administration of TGF‐β1 compared to control (Figure 3c). *Fst* expression was increased in myotubes treated with siAcvr1b in the absence of cytokines (*p* < 0.000). Gene expression levels of E3 ubiquitin ligases *Trim63* and *Fbxo32* were not different after knockdown of either *Acvr1b* or *Tgfbr1* (Figure 3d,e), suggesting impaired myotube growth was not due to an increased protein breakdown. Gene expression of *Myh3*, a gene that codes for embryonic MHC isoform, showed a mild increase by siRNA targeting *Acvr1b* without cytokines (*p* = 0.012) and by siRNA targeting both *Acvr1b* and *Tgfbr1* in the presence of TGF‐β1 (*p* = 0.018) and myostatin (*p* = 0.014), suggesting improved myogenesis by interference with both receptors (Figure 3f).

As TGF‐β1 is a pro‐fibrotic cytokine in muscle stem cells (Hillege et al., 2020; Shi et al., 2021), we next investigated the expression levels of genes related to fibrosis. *Tgfb1* expression levels were decreased by the knockdown of both *Tgfbr1* and *Acvr1b* compared to the knockdown of *Acvr1b* (*p* = 0.003) or control in the presence of TGF‐β1 (*p* < 0.000) (Figure 3g). Gene expression of *Ccn2*, coding for connective tissue growth factor (CTGF), was increased by 10‐fold in myotubes exposed to TGF‐β1 (*p* < 0.000) and twofold in myotubes exposed to myostatin (*p* = 0.001) (Figure 3h). After administration of either TGF‐β1 (*p* = 0.001) or myostatin (*p* = 0.003), gene expression of *Ccn2* was decreased in myotubes cultured with siRNA targeting both *Acvr1b* and *Tgfbr1*. In addition, gene expression of *Col1a1* was significantly reduced in myotube with siRNA targeting both *Acvr1b* and *Tgfbr1* with supplement of TGF‐β1 (*p* = 0.025) (Figure 3i).

Fusion is essential to form multinucleated myotubes. Myomerger (*Mymx*) and Myomaker (*Mymk*) are responsible to myoblasts fusion (Chen et al., 2020). For myotubes treated with TGF‐β1, *Mymk* expression level was increased when cells were exposed to si*Tgfbr1* compared to those being exposed to siControl and siAcvr1b (*p* = 0.034)*.* For myotubes stimulated by myostatin, *Mymk* expression was increased when they were exposed to siRNA targeting *Tgfbr1* (*p* = 0.006) and both *Acvr1b* and *Tgfbr1*(*p* = 0.033) compared to myotubes being exposed to siAcvr1b. In contrast, *Mymx* expression was significantly decreased without (*p* = 0.014) or with TGF‐β1 (*p* = 0.01) or myostatin (*p* = 0.002) in myotubes exposed to siAcvr1b and siTgfbr1 (Figure 3j,k).

### 3.4 Knockdown of TGF‐β type I receptors increases expression level of dystrophin‐related gene Sntb1

Based on the finding that regulatory region of chromatin targeted by TGF‐β signalling‐regulated Smad includes syntrophin‐associated serine/threonine kinase (Wang et al., 2005), we investigated whether interference with TGF‐β signalling pathway disrupted syntrophin expression. Our results show that targeting *Tgfbr1* (*p* < 0.001) or both type I receptors (*p* < 0.001) by siRNA increased *Sntb1* expression regardless of supplement of cytokines (Figure 3l). However, expression levels of α1‐syntrophin (*Snta1*) were not affected (Figure 3m). Therefore, the knockdown of both TGF‐β type I receptors in myotubes stimulated gene expression of *Sntb1* which encodes a subunit of DGC component.

### 3.5 Knockdown of Sntb1 increases myotube diameter by increasing muscle fusion

**Figure 4:**
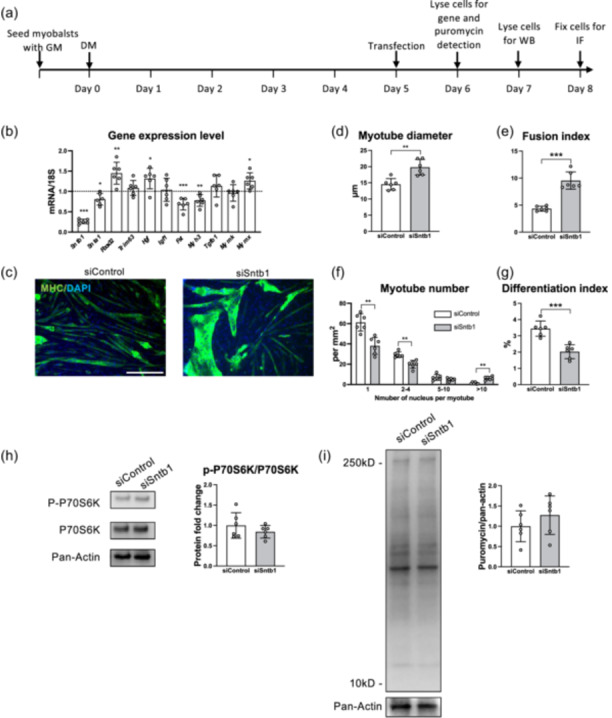
Knocking down *Sntb1* increases myotube fusion. (a) Scheme of cell culture and siRNA interference within myotube after differentiation for 5 days. (b) Gene expression of protein turnover and cell differentiation within myotubes transfected by siSntb1 for 24 h compared to myotubes transfected by siControl. (c) Myotubes with more nuclei were found in myotube transfected by siSntb1 by immunofluorescent staining of myosin heavy chain (MHC) (green) and nuclei (DAPI, blue). Scale bar = 250 µm. (d) Myotube diameter and (e) fusion index were increased in myotubes transfected by siSntb1. (f) More myotube with more than 10 nuclei were found when cells that were transfected by siSntb1. (g) The differentiation index was decreased in myotubes treated with siSntb1. Data are presented as mean ± SD. *p < 0.05, **p < 0.01, ***p < 0.001. *N* = 6. Data were analysed by independent *t*‐tests.

Given that the increased expression level of *Sntb1* was associated with decreased myotube diameter, we sought to elucidate the effect of *Sntb1* on muscle differentiation and hypertrophy. To this end, *Sntb1* in myotubes was knocked down by siRNA (Figure 4a). When *Sntb1* expression level was significantly reduced by 70% compared to siControl (*p* < 0.000), *Snta1* expression was decreased by about 20% (*p* = 0.011). The expression levels of *Fbxo32* (*p* = 0.005), *Hgf* (*p* = 0.014) and *Mymx* (*p* = 0.011) were increased, while those of *Myh3* (*p* = 0.007) and *Fst* (*p* = 0.001) were decreased (Figure 4b). Expression levels of *Igf1* (*p* = 0.841) and *Mymk* (*p* = 0.671) were not changed. Increased *Fbxo32* has been shown to inhibit protein synthesis signalling and stimulate proteolysis (Sacheck et al., 2004; Wang et al., 2010), suggesting that *Sntb1* downregulation may cause myotube atrophy. However, since siSntb1 stimulated *Hgf* expression, this atrophic effect could have been antagonized by the increased expression level of HGF, as this growth factor has been shown to activate the AkT/mTOR signalling (Perdomo et al., 2008). Moreover, myotube diameter was significantly increased after *Sntb1* knockdown (*p* = 0.002) (Figure 4c,d), accompanied by an increased fusion index (*p* < 0.000) (Figure 4e). The increase in fusion may have contributed to increased myotube diameter (Figure 4f). As a result, increased myotube fusion resulted in a reduced differentiation index (Figure 4g). Noteworthy, p‐P70S6K/P70S6K ratio and global protein synthesis rate measured by the SUnSET assay was not increased by knocking down *Sntb1* (Figure 4h,i). We therefore conclude that myotube hypertrophy by targeting *Sntb1* was caused by accelerated nuclear accretion within myotubes rather than by elevated protein translation rate.

## 4 DISCUSSION

**Figure 5:**
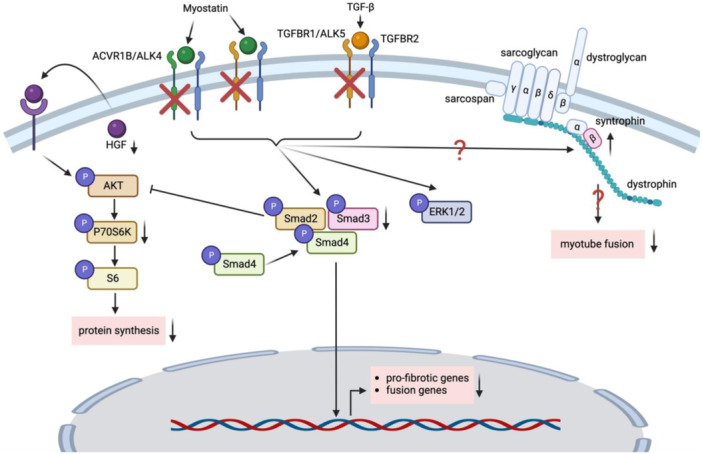
Schematic summarizing the mechanisms via which myotube size and myoblast fusion are regulated by ACVR1B and TGFBR1. When both type I receptors ACVR1B and TGFBR1 were knocked down, myostatin or TGF‐β1‐induced Smad2/3 signalling pathways were downregulated. Reduced Smad2/3 signalling decreased pro‐fibrotic gene transcription. Although the inhibitory effect of Smad2/3 signalling on the AKT/P70S6K/S6 signalling pathway was mitigated, phosphorylation of P70S6K/S6 was not upregulated, which may be attributed to reduced expression of HGF. β1‐syntrophin was upregulated upon simultaneous type I receptors knockdown while myoblasts fusion was decreased, resulting in reduced myotube diameter. TGF‐β, transforming growth factor‐β.

The TGF‐β superfamily plays a pivotal role in the regulation of muscle mass. In this study, we show that targeting either *Acvr1b* or *Tgfbr1* in vitro caused modest morphological and transcriptome changes in cultured myotubes, indicating a functional redundancy of these receptors in inducing TGF‐β signalling. In contrast, simultaneous knockdown of *Acvr1b* and *Tgbfr1* impaired myotube growth and reduced protein synthesis signalling. We show that knockdown of *Acvr1b* and *Tgfbr1* in myotubes in vitro has different effects compared to combined receptors knockout in skeletal muscle in vivo (Hillege et al., 2022). In addition, simultaneous knockdown of *Acvr1b* and *Tgbfr1* reduced expression levels of pro‐fibrotic and fusion genes. We further showed that *Hgf* expression was reduced, while *Sntb1* expression was increased. In contrast, knocking down *Sntb1* expression increased myotube diameter, nuclear accretion and *Hgf* expression, suggesting critical role of endogenous *Hgf* and *Sntb1* on the regulation of muscle cell size (Figure 5). These results show that direct and acute downregulation of TGF‐β type I receptors within myotube reduce cell size and protein synthesis signalling.

### 4.1 Differences between two TGF‐β type I receptors ACVR1B and TGFBR1

We attempted to assess different effects on myotube size by ACVR1B and TGFBR1 in response to TGF‐β1 and myostatin in the regulation of muscle mass in vitro. TGF‐β1 is a potent cytokine for fibrosis (Meng et al., 2016), which stimulates collagen mRNA and protein production in muscle stem cells (Shi et al., 2021), but curbes myoblast fusion (Girardi et al., 2021). Myostatin is known for its pro‐fibrotic effect and inhibitory impact on muscle cell differentiation (Hillege et al., 2020). Our data indicate that blocking *Acvr1b* solely has little effect on myotube morphological and transcriptome change regardless of the administration of TGF‐β1 or myostatin. The current results demonstrate that TGF‐β1 enhances pro‐fibrotic transcripts more substantially than myostatin. Knockdown of *Tgfbr1*, rather than *Acvr1b*, decreased transcription of pro‐fibrotic genes *Ccn2*, *Tgfb1* in the presence of TGF‐β1 (Figure 3g,h). Expression of *Col1a1* was not decreased by blocking *Acvr1b* but by knockdown of both *Acvr1b* and *Tgfbr1* with TGF‐β1 (Figure 3i). These results indicate that TGFBR1 is the specific regulator of fibrogenesis in muscle cells. Besides, the knockdown of *Tgfbr1*, but not *Acvr1b*, decreased the expression level of *Fst*, which ameliorated the inhibitory effect on myostatin and activin A. This suggests that TGFBR1 plays a key role in the regulation of the activity of activins and myostatin. For myoblast differentiation, antisense oligonucleotides‐mediated *Tgfbr1* exon skipping has been shown to stimulate differentiation of myoblasts (Kemaladewi et al., 2014). However, here we show that targeting neither *Tgfbr1* nor *Acvr1b* individually altered fusion, differentiation index and Smad2/3 phosphorylation in differentiated myotubes. This may be explained by the increased *Acvr1b* expression upon sole knockdown of *Tgfbr1* (Figure 1b), suggesting the functional redundancy of these type I receptors in the regulation of myofibre differentiation and growth. Taken together, ACVR1B and TGFBR1 individually stimulate TGF‐β canonical signalling in myotubes and TGFBR1 is required for pro‐fibrotic gene expression.

### 4.2 Simultaneous downregulation of type I receptors in myotube reduces protein synthesis signalling

Overexpression of TGF‐β and myostatin in skeletal muscle has been shown to cause muscle wasting disorders (Ábrigo et al., 2018; Klein, 2022), reduce muscle force‐generating capacity (Lozier et al., 2018) and induce muscle fibrosis (Narola et al., 2013). In a series of in vivo studies, it has been shown that the inhibition of TGF‐β signalling pathway in vivo by either knockout of TGF‐β receptors (Lee et al., 2020) or transcriptional factors Smad2/3 (Umezu et al., 2021), or by using a neutralizing antibody of TGF‐β type II receptors (Morvan et al., 2017), have shown to increase muscle mass, strength and alleviate muscle fibrosis. Our recent study shows that targeting both type I receptors in vivo increases muscle mass by increasing signalling for protein synthesis and inhibiting catabolic activity (Hillege et al., 2022). Unexpectedly, under the current experimental conditions in vitro, knockdown of *Acvr1b* and *Tgfbr1* in myotubes reduced cell diameter (Figure 1e).

Findings of the impaired muscle growth by blocking TGF‐β type I or II receptors in vitro challenged the idea of how TGF‐β signalling regulates muscle mass. TβRII expression was increased during myoblast differentiation. Inhibition of TβRII in C2C12 cells reduces AKT phosphorylation and protein synthesis (Li et al., 2023). In addition, TGF‐β receptor type I expression has been shown to increase during skeletal muscle differentiation in vitro as well (Droguett et al., 2010). Inhibition of TGF‐β receptor type I by SB 431542 inhibits myosin expression and myotube formation, suggesting the importance of TGF‐β receptors during muscle differentiation (Droguett et al., 2010). Moreover, the inhibition of ACVR1B and TGFBR1 by SB 431542 inhibited phosphorylation of P70S6K after 48 h (Watt et al., 2010). In line with other studies, our results show that knockdown of TGF‐β type I receptors reduced myotube size and phosphorylation of P70S6K in the presence of myostatin, suggesting the positive role of TGF‐β type I receptors and TGF‐β signalling in muscle growth.

The smaller cell size likely resulted from a reduced protein synthesis signalling, while the rate of protein degradation was likely not affected upon gene knockdown (i.e., *Trim63* and *Fbxo32* expression levels did not change) (Figures 1d,e and 3d,e). Since phosphorylated levels of AKT were not affected, reduced P70S6K phosphorylation may be due to reduced phosphorylation of mTOR1 through an AKT‐independent manner (Bahrami‐B et al., 2014; Egerman & Glass, 2014). Phosphorylated mTOR1 enhances the rate of protein synthesis by activating S6 through phosphorylation of P70S6K and by phosphorylation of eukaryotic translation initiation factor 4E binding protein B 1 (4E‐BP1) (Xu et al., 2010). When 4E‐BP1 is hyperphosphorylated, it dissociates from eIF‐4E, leading to the initiation of translation. In contrast, hypophosphorylated 4E‐BP1 strongly binds to eIF‐4E, leading to the repression of translation. It is conceivable that the reduced phosphorylation of mTOR1 may have decreased the phosphorylation of 4E‐BP1, resulting in a reduced rate of protein synthesis.

### 4.3 Myotube size is reduced by knockdown of both receptors despite reduced Smad2/3 signalling

Our study demonstrated that Smad2/3 signalling was inhibited by the downregulation of *Acvr1b* and *Tgfbr1* expression, while cell size did not increase. The underlying mechanism is unclear. One potential explanation is that inhibiting Smad2/3 signalling and downregulation of both type I receptors in the remaining myoblasts may negatively affect myoblasts differentiation and fusion. A previous study has shown that Smad3‐null mice showed impaired satellite cell self‐renewal function and severe muscle wasting (Ge et al., 2011). Knockout of Smad2 expression in primary myoblasts in vitro resulted in decreased myotube size and loss of Smad2 in MuSCs in vivo resulted in impaired regeneration upon acute injury (Lamarche et al., 2021). Administration of TGFBR1 inhibitor inhibited myosin expression and myotube formation in vitro, suggesting the critical role of TGFBR1 at the last step of muscle differentiation (Droguett et al., 2010). Indeed, the fusion index (Figure 1f) tended to be lower and *Mymx* expression was significantly reduced in myotubes lacking both type I receptors (Figure 3k), suggesting diminished myoblasts fusion capacity. Therefore, our data imply that TGF‐β signalling initiated by ACVR1B and TGFBR1 plays an indispensable role in the regulation of cell fusion (Girardi et al., 2021).

Another possible factor causing impaired myotube growth by knocking down both type I receptors may work in a Smad2/3‐independent manner. The impaired myotube growth may be attributed to the decreased *Hgf* expression. HGF enhances muscle protein synthesis signalling pathway and increases protein expression of myogenic factors in vivo (Hauerslev et al., 2014). Intraperitoneal injection of HGF in mice caused activation of the AKT/mTOR/P70S6K protein synthesis pathway as well as decreased expression of *Trim63* and *Fbxo32* (Hauerslev et al., 2014). In L6 myotubes and mouse C2 cells, HGF stimulates the phosphorylation of AKT (Elia et al., 2007; Perdomo et al., 2008). Inhibition of HGF expression decreased the size of newly formed myofibres during muscle regeneration after muscle injury (Choi et al., 2019). We have shown that the knockout of both type I receptors induced muscle hypertrophy in fast‐type muscle expressed more *Hgf* 35 days and 3 months after gene knockout (Hillege et al., 2022; Shi et al., 2023). The lower *Hgf* expression may contribute to the diminished activation of the AKT/mTOR/P70S6K/S6 signalling pathway. Indeed, we speculate that reduced *Hgf* expression in myotubes decreased protein synthesis which might offset the stimulation of protein synthesis caused by inhibition of Smad2/3 and therefore reduced myotube diameter. The striking contrast in the effects of combined TGF‐β receptor knockout in myofibres in vivo, and knockdown in myotubes in vitro suggests the critical involvement of growth factors, such as HGF, and the interaction of non‐myogenic cells in the stimulation of muscle hypertrophy.

### 4.4 Myotube size is regulated by β1‐syntrophin

In addition, another factor that likely contributed to the reduction in myotube size is the increased expression level of *Sntb1*. *Sntb1* expression was downregulated by TGF‐β1, but not by myostatin. Knockdown of *Tgfbr1* solely was sufficient to upregulate *Sntb1* expression. This indicates that of both receptors, TGFBR1 was mostly involved in the regulation of *Sntb1* expression compared to ACVR1B. Myod and myogenin are the transcriptional activators for the *Sntb1* gene (Hamed et al., 2017). Given the inhibitory effect of Smad3 on the function of Myod (Langley et al., 2002), the trend of reduced p‐Smad3 in myotubes in the current study by knocking down both *Acvr1b* and *Tgfbr1*, may explain the increased expression of *Sntb1*.

β1‐syntrophin is a DGC unit that anchors muscle cells to ECM and interacts with actin (Valera et al., 2021). Syntrophin binds to F‐actin and inhibits actin‐activated myosin ATPase activity to interrupt the interaction between actin and myosin (Iwata et al., 2004). F‐actin foci were present at the fusion site of myoblast (Guerin & Kramer, 2009) and a robust, cortical F‐actin wall was aligned in differentiating myoblasts before fusion (Duan & Gallagher, 2009). Increased SNTB1 may reconstruct myoblasts cytoskeleton, interrupt the assembly of cytoskeleton proteins and inhibit myoblasts fusion. The expression level of *Sntb1* was reduced in hypertrophic muscle lacking both type I receptors in vivo (Shi et al., 2023), we expected that combined knockdown of type I receptors in vitro reduced *Sntb1* expression while enhanced myotube size. In contrast, *Sntb1* was upregulated in myotubes, accompanied by a reduction in diameter (Figure 3l). Increased myotube diameter induced by knockdown of *Snbt1* was attributed to increased myoblasts fusion rather than to an increase in protein synthesis rate (Figure 4i). This suggests that *Sntb1* is likely to be the regulator of muscle cell size through inhibition of myoblasts fusion.

Very little is known about the role of the DGC on skeletal muscle development and growth. Upon knockdown of *Sntb1* in myotubes, fusion index and *Mymx* expression levels were increased, but phosphorylation of P70S6K was not changed, suggesting an important regulatory role of *Sntb1* on myoblasts fusion. Indeed, a previous study showed that increased muscle mass was found in mice lacking α1‐syntrophin (Hosaka et al., 2002), which was independent of P70S6K‐mediated protein synthesis. Moreover, the lack of α‐, β1‐ and β2‐syntrophin increased the thickness of the left ventricular posterior wall in mice, suggesting its role in cardiac muscle hypertrophy (Kim et al., 2019). These findings suggest that disrupting β1‐syntrohin in myofibres increases the muscle cell size. In addition, since β1‐syntrohin has been detected predominantly in human myofibers expressing neonatal MHC and its expression level declined by 5 months (Compton et al., 2005), increased *Sntb1* expression in myotubes with simultaneous receptor knockdown may contribute to the slower differentiation rate of myotubes. Overall, the regulatory role of DGC units to the cytoskeletal organization and muscle mass in myotubes lacks a molecular underlining mechanism and needs further research.

## 5 CONCLUSION

This study shows that simultaneous knockdown of *Acvr1b* and *Tgfbr1* in differentiated C2C12 myotubes in vitro reduces phosphorylation of Smad2/3 and myotube diameter, while individual interference with either type I receptors has little effect. Reduced myotube diameter is likely attributed to decreased protein synthesis signalling as phosphorylation of P70S6K is reduced and is associated with reduced expression of *Hgf*. While gene expression of protein degradation is not altered. An increase in *Sntb1* expression by simultaneous knockdown of *Acvr1b* and *Tgfbr1* is associated with reduced myotube size. Blocking *Sntb1* expression increased myotube diameter by stimulating cell fusion but did not affect protein translation rate in myotubes. These results indicate that the direct effects of the knockdown of type I receptors in myotube is the reduction in myotube cell size, which is regulated by SNTB1.

## AUTHOR CONTRIBUTIONS

**Andi Shi**: Conceptualization; data curation; investigation; methodology; project administration; writing original draft; writing review and editing; visualization. **Chuqi He**: Data curation; investigation; methodology; project administration. **Kirsten Otten**: Data curation; investigation; project administration. **Gang Wu**: Supervision; writing review and editing. **Tymour Forouzanfar**: Supervision; **Rob C. I. Wüst**: Supervision; conceptualization; writing review and editing. **Richard T. Jaspers**: Conceptualization; supervision; funding acquisition; writing review and editing.

## CONFLICT OF INTEREST STATEMENT

The authors declare no conflict of interest.

## Supporting information

Supporting information.
