# Supplementary material for: Reduced myotube diameter induced by combined inhibition of transforming growth factor‐β type I receptors Acvr1b and Tgfbr1 is associated with enhanced β1‐syntrophin expression
Source: J Cell Physiol. 2024 Aug 20;239(12):e31418. doi: 10.1002/jcp.31418 (PMC11649968; doi:10.1002/jcp.31418)
Supplement: Supplementary file 1 — Supporting information. [file JCP-239-0-s001.docx]

Appendices

Supplementary Table 1. Sequence of primers for qPCR

| Gene | Forward | Reverse |
| --- | --- | --- |
| *18S* | GTAACCCGTTGAACCCCATT | CCATCCAATCGGTAGTAGCG |
| *Acvr1b* | TGCTGCGCCATGAAAACATC | TGCCCACAATCTCCATATGCA |
| *Tgfbr1* | CCTCGAGACAGGCCATTTGT | AGACGAAGCAGACTGGACCA |
| *Tgfb1* | GCTGACCCCCACTGATACG | CCTGTATTCCGTCTCCTTGGTT |
| *Ccn2* | CCACCCGAGTTACCAATGAC | GCTTGGCGATTTTAGGTGTC |
| *Col1a1* | ATGTTCAGCTTTGTGGACCT | CAGCTGACTTCAGGGATGT |
| *Hgf* | GATTATTGCCCTATTTCCCGTTGTG | TGGCACAGGATATTACAGGATGG |
| *Myh3* | CGCAGAATCGCAAGTCAATA | CAGGAGGTCTTGCTCACTCC |
| *Igf1ea* | GTGTTGCTTCCGGAGCTGTG | CAATGTACTTCCTTCTGAGTC |
| *Trim63* | CGTCCAGAGCGTGTGTCTCACTC | GGGCTACCTTCCTCTCAAGTGC |
| *Fbxo32* | AGACTGGACTTCTCGACTGC | TCAGCTCCAACAACAGCCTTACT |
| *Sntb1* | AGTGCTGCTGGAAGTGAAGTAT | ATCCCAATCTCAGACACCGGG |
| *Snta1* | AGAAGTGTCTACAGCCTGCAC | GTGAAGCCCTTGTCGATGTG |
| *Fst* | TGAGAAAGGCCACCTGCTT | GACACAGCTCATCGCAGAGA |
| *Mymk* | TCATTGCTGTAAAGTGGCTGAAG | TGGGTGTAGATGCTCTTGTCG |
| *Mymx* | GCAGGCAAAAGCATCCTGAA | CAGGACCTGCACCAGTTCTA |

Supplementary Table 2. Antibodies used for Westernblot

| Antibody | Dilution | Antibody type, Catalogue number, Manufacturer |
| --- | --- | --- |
| Phospho-Smad2 (Ser465/467) (138D4) | 1:1000 | Rabbit mAb, 3108, Cell Signaling Technology, Massachusetts, USA |
| Phospho-Smad3 (Ser423/425) (C25A9) | 1:1000 | Rabbit mAb, 9520, Cell Signaling Technology, Massachusetts, USA |
| Smad2/3 | 1:1000 | Mouse IgG1, κ, 610843, BD Biosciences, New Jersey, USA |
| Phospho-AKT (Ser473) | 1:1000 | Rabbit mAb, 4060, Cell Signaling Technology, Massachusetts, USA |
| AKT (pan) (C67E7) | 1:2000 | Rabbit mAb, 4691, Cell Signaling Technology, Massachusetts, USA |
| Phospho-p70S6 Kinase (Thr389) (108D2) | 1:2000 | Rabbit mAb, 8209, Cell Signaling Technology, Massachusetts, USA |
| p70S6 Kinase (49D7) | 1:2000 | Rabbit mAb, 2708, Cell Signaling Technology, Massachusetts, USA |
| Phospho-S6 Ribosomal Protein (S235/236) | 1:2000 | Rabbit Ab, 2211S, Cell Signaling Technology, Massachusetts, USA |
| S6 Ribosomal Protein (5G10) | 1:4000 | Rabbit mAb, 2217S, Cell Signaling Technology, Massachusetts, USA |
| Phospho-MAPK (Erk1/2) (Thr202/Yyr204) (D13.14.4E) XP^®^ | 1:1000 | Rabbit Ab, 4370S, Cell Signaling Technology, Massachusetts, USA |
| p44/42 MAPK (Erk1/2) (137F5) | 1:4000 | Rabbit mAb, 4695, Cell Signaling Technology, Massachusetts, USA |
| Puromycin | 1:2000 | Mouse mAb, clone 12D10, Sigma-Aldrich, Germany |
| Pan-Actin | 1:2000 | Rabbit Ab, 4968, Cell Signaling Technology, Massachusetts, USA |
| Rabbit IgG, HRP | 1:4000 | Goat Ab, P0448, Agilent Dako, USA |
| Mouse IgG (H+L), HRP | 1:4000 | Rabbit Ab, 31457, Thermo Fisher Scientific |
